# A randomized controlled trial of the effectiveness of Housing First in a small Canadian City

**DOI:** 10.1186/s12889-019-7492-8

**Published:** 2019-08-22

**Authors:** Tim Aubry, Jimmy Bourque, Paula Goering, Susan Crouse, Scott Veldhuizen, Stefanie LeBlanc, Rebecca Cherner, Paul-Émile Bourque, Sarah Pakzad, Claudette Bradshaw

**Affiliations:** 1 School of Psychology & Centre for Research on Educational and Community Services, University of Ottawa, Vanier Hall #5018, Ottawa, ON K1N 6N5 Canada; 2 Centre de recherche et de développement en éducation, Faculté des sciences de l’éducation, Université de Moncton, Moncton, NB Canada; 3 Centre for Addiction and Mental Health & Department of Psychiatry, University of Toronto, Toronto, ON Canada; 4 Salvus Clinic, Moncton, NB Canada; 5 Centre for Addictions and Mental Health, Toronto, ON Canada; 6 École de psychologie, Université de Moncton, Moncton, ON Canada; 7 Mental Health Commission of Canada, Ottawa, ON Canada

**Keywords:** Homelessness, Housing first, Assertive community treatment

## Abstract

**Background:**

The paper presents two-year findings from a study investigating the effectiveness of Housing First (HF) with assertive community treatment (ACT) in helping individuals with serious mental illness, who are homeless or precariously housed and living in a small city, to become stably housed.

**Methods:**

The research design was a parallel group non-blinded RCT with participants randomly assigned after the baseline interview to receive HF with ACT (*N* = 100) or treatment as usual (TAU; *N* = 101). Participants were interviewed every 3 months over 21/24 months to investigate changes on a range of housing and psychosocial outcomes. The primary outcomes were housing stability (as defined by a joint function of number of days housed and number of moves) and improvement in community functioning. Secondary predicted outcomes were improvements in self-rated physical and mental health status, substance use problems, quality of life, community integration, and recovery.

**Results:**

An intent-to-treat analysis was conducted. Compared to TAU participants, HF participants who entered housing did so more quickly (23.30 versus 88.25 days, *d* = 1.02, 95% CI [0.50–1.53], *p* < 0.001), spent a greater proportion of time stably housed (*Z* = 5.30, *p* < 0.001, *OR* = 3.12, 95% CI [1.96–4.27]), and rated the quality of their housing more positively (*Z* = 4.59, *p* < 0.001, *d* = 0.43, 95% CI [0.25–0.62]). HF participants were also more likely to be housed continually in the final 6 months (i.e., 79.57% vs. 55.47%), χ^2^ (2, *n* = 170) = 11.46, *p* = .003, Cramer’s V = 0.26, 95% CI [0.14–0.42]). HF participants showed greater gains in quality of life, (*Z* = 3.83, *p* < 0.001, ASMD = 0.50, 95% CI [0.24–0.75]), psychological integration (*Z* = 12.89, *p* < 0.001, pooled ASMD = 0.91, 95% CI [0.77–1.05]), and perceived recovery (*Z* = 2.26, *p* = 0.03, ASMD = 0.39, 95% CI [0.05–0.74]) than TAU participants.

**Conclusions:**

The study indicates that HF ends homelessness significantly more rapidly than TAU for a majority of individuals with serious mental illness who have a history of homelessness and live in a small city. In addition, compared to TAU, HF produces psychosocial benefits for its recipients that include an enhanced quality of life, a greater sense of belonging in the community, and greater improvements in perceived recovery from mental illness.

**Trial registration:**

International Standard Randomized Control Trial Number Register Identifier: ISRCTN42520374, assigned August 18, 2009.

## Background

In line with other Western countries, homelessness has emerged over the past 30 years as a significant social problem in Canada [1]. A significant proportion of the homeless population is comprised of single men and women with serious mental illness who experience ongoing housing difficulties and homelessness [2–4].

The problem of chronic homelessness among people with serious mental illness appeared irresolvable for many until the appearance of a new approach to combining housing and support, known as Housing First (HF) [5]. The new approach, developed by a community agency in New York City known as Pathways to Housing, involved moving individuals into regular housing in the community as rapidly as possible without any pre-conditions [6]. Hence, the name of “Housing First,” in contrast to more traditional approaches that could be referred to as “Treatment First,” wherein individuals are required to engage in treatment in order to stabilize their functioning prior to being considered able to live independently in regular housing.

An important component of HF entails providing individuals with rent supplements so that they are able to have some choice in their accessing of private market rental housing [7]. In addition to assistance with finding, moving into, and furnishing a new home, HF services include portable and intensive community support delivered by an Assertive Community Treatment (ACT) team or through Intensive Case Management (ICM). Other aspects of the HF approach include an emphasis on consumer choice and empowerment, harm reduction, community integration, and recovery [6]. A recent review of the research on permanent supportive housing, including Pathways HF, assessed the level of evidence of its effectiveness as “moderate” [8].

The first line of research on Pathways HF with ACT included two RCTs conducted in New York City. Both trials found that individuals receiving Pathways HF achieved better housing outcomes than those receiving residential continuum model services [9] or standard care [10], with HF recipients leaving homelessness sooner with a higher proportion becoming stably housed. Three quasi-experimental studies also found that individuals receiving Pathways HF with ACT experienced better housing outcomes than individuals receiving either residential continuum services [5, 11] or standard care [12] as well as decreased use of inpatient and emergency department services, decreased involvement in the justice system, improved quality of life compared to outpatient mental health services [13] and a greater decrease in substance use relative to standard care [12, 14].

In the context of the promising early findings in these initial studies, the Mental Health Commission of Canada, funded by Health Canada, launched in 2008 the At Home / Chez Soi (AHCS) Demonstration Project, a pragmatic RCT in five Canadian cities, that was intended to test the effectiveness of Pathways HF in addressing homelessness among people with serious mental illness in comparison to treatment as usual (TAU) [15]. The trial tested two variants of HF with individuals with two different levels of need. People with a high level of need received HF with ACT. In contrast, people with a moderate level of need were supported in the HF program with ICM. In addition, each site also delivered a program that involved an adaptation for the local homeless population: single-site supportive housing in the Vancouver site, HF adapted for an Indigenous population in the Winnipeg site, HF adapted for a multi-cultural population in the Toronto site, a combination of HF and supported employment in the Montreal site, and a rural arm in the Moncton site.

Although there were adaptations of HF in the different sites, correspondence to the approach was ensured by the provision of initial intensive training and ongoing technical support by Pathways to Housing personnel. As well, two implementation evaluations and two assessments of program fidelity were conducted at all the sites during the project [16, 17]. The published study protocol provides a detailed description of the study’s methods [15].

A large number of publications have been produced from the AHCS Demonstration Project over the past several years. Parallel with the initial Pathways to Housing studies, HF combined with ACT for people with high needs produced a large effect relative to TAU in moving people out of homelessness and into stable housing [18, 19]. HF recipients with high needs reported greater improvements in quality of life than participants receiving standard care at 12 months [18]. However these differences in quality of life between the two groups were no longer present at 24 months as participants receiving TAU experienced improvement in the second year of the trial, catching up to HF participants [19]. HF recipients with high needs showed a similar pattern of change in community functioning with greater improvement compared to TAU by 12 months [18], but again the differences disappeared by 24 months [19].

Site-specific findings have been published, confirming the superior housing outcomes for HF with ACT relative to TAU but also showing HF recipients experiencing greater improvements on non-housing outcomes including some not evident in the multi-site findings. In Vancouver, individuals receiving HF with ACT reported significantly greater improvements in their overall quality of life and quality of life associated with their living situation and safety after 6 months and 12 months [20]. Also individuals with high needs receiving HF with ACT had a significantly lower number of criminal convictions than recipients of TAU [21]. At the site of the current study, previous research comparing HF and TAU recipients on use of health services showed HF recipients experiencing more days of hospitalizations for psychiatric problems over the course of the 2 year study [22]. These findings were interpreted as being the result of the intensive support provided by the ACT team that includes facilitating the access to psychiatric hospital treatment when it is needed.

Table 1 presents the program logic model for HF programs with ACT in the AHCS project [7]. As shown in the program logic model, the receipt of HF services is expected to assist an individual to rapidly exit homelessness and establish housing stability in the first 6 months. Improvements in health status, quality of life, and community integration ensues, ultimately leading longer-term to recovery [6, 7]. Relative to the other sites in the bigger cities in AHCS, individuals receiving TAU in Moncton used less social services (i.e., supportive housing, help lines, day centres) and less health care (i.e., substance use treatment, hospital stays for psychiatric treatment, emergency department visits) [23]. Moreover, publicly funded mental health services were relatively limited and traditional in nature, made up of outpatient and day program services in community health centres and hospitals, and psychiatrists in private practice [24].

**Table 1:** Program Logic Model for Pathways Housing First Program

Domain/ Inputs	Theoretical Principles	Program Activities	Outcomes		
			Immediate (0–6 months)	Medium-term (6–24 months)	Long-term (>2 years)
Housing/ Rent supplements	Housing choice and community integration	-Assess consumer housing preferences -Rapid housing procurement -Permanent housing -Obtain rent supplement -Assistance with furnishing housing -Typically scattered-site housing, but depends on consumer choice -Lease with private landlord	-Rapidly housed in place of choice -Reduced contact with non-supportive contacts - Development of new relationships with landlords and neighbours	-Increased housing stability -Reduced homelessness -Increased housing choice -Increased quality of housing -Increased housing satisfaction -Positive relationships with landlords, neighbours, and other community members	-Increased housing stability -Reduced homelessness -Maintenance of housing choice, quality, and satisfaction even if housing changes -Maintenance of relationships with landlords, neighbours, and other community members
Services/ ACT or ICM services	Separation of housing and services	-Mobile ACT outreach	-Development of working alliance with ACT staff	-Maintenance of working alliance with staff	-Maintenance of working alliance with staff -Increased community integration
	Services based on choice, recovery-orientation, and community integration	Service philosophy -Staff values of choice and recovery -Assertive engagement -Assess consumer interests (e.g., work, education, social, family) -Assist consumer in accessing public benefits and health services -Harm reduction -Individualized consumer-centered planning -Broad range of goals	-Increased participation in mental health treatment -Increased participation in substance use treatment -Increased access to public benefits and health services -Reduced use of hospital and emergency services -Reduced involvement in criminal justice system	-Maintenance of reduced use of hospital and emergency services and involvement in criminal justice system -Improved community functioning -Increased subjective quality of life -More positive consumer narratives - Development of future-focused orientation -Improved clinical outcomes (i.e., reduced psychiatric symptoms and substance use)	-Maintenance of reduced use of hospital and emergency services and involvement in criminal justice system -Maintenance of community functioning, subjective quality of life, and consumer narratives -Increased involvement in work or education
		Service array -Housing -Psychiatric services -Primary care -Social integration services			
		Program structure -Weekly visits -Team meetings -Low consumer:staff ratio -Peer specialist			

The primary predicted outcomes for the assessment of effectiveness of HF were the achievement of housing stability (as defined by a joint function of number of days housed and number of moves) and improvement in community functioning (i.e., level of ability to live independently in the community) [15]. Secondary predicted outcomes were improvements in self-rated physical and mental health status, substance use problems, quality of life, community integration, and recovery [15]. It was hypothesized that HF would produce greater improvements on primary and secondary outcomes than TAU.

In summary, previous research on HF has demonstrated its effectiveness in ending homelessness for a majority of people with severe and persistent mental illness in large cities in North America. In addition, some studies have also shown recipients of HF to report greater improvements in their quality of life compared to individual receiving standard care. This current article presents the results from the Moncton site of the AHCS project. The study of this site is original as it investigates the outcomes of HF with ACT in a small city, a context in which there has been no previous research on its effectiveness to date.

It is unknown if HF participants located in a small city will derive different and greater benefits than those found to date in large North American cities. The catchment area of the site was the tri-city Moncton, Riverview, and Dieppe in the province of New Brunswick. The population of the tri-city was approximately 139,000 at the time of the study [25]. At the beginning of AHCS project in Moncton, there were 640 individuals who were homeless and who stayed in emergency shelters in the tri-city [26].

Another unique aspect of the study at the Moncton site, relative to the other sites in the AHCS project, involved the delivery of HF with ACT to individuals with either moderate or high needs. Moncton was the only site in the AHCS project to provide community support from an ACT team to individuals with moderate needs. In line with ACT, the community support entailed home visits and a range of individual services that included life skills training, counselling, crisis intervention, and referral to health and social services.

## Methods

### Study design

The research design for the Moncton site was a parallel-group non-blinded RCT with participants randomly assigned after the baseline interview to receive HF with ACT or TAU. In the four other cities of the AHCS trial, only high need participants received ACT, with moderate need participants receiving ICM. However, at the Moncton site, the relatively small homeless population resulted in only one HF program being tested; this program provided ACT to all participants, regardless of need level. Ethical approval for the study was received from the IRBs of the Université de Moncton and the University of Ottawa and the two Regional Health Authorities (i.e., Horizon and Vitalité) involved with the project. Participants provided signed informed consent before participating in the screening process and before the first interview. None of the authors have any competing interests.

### Participants

Participants were recruited through referrals from personnel located in community agencies and institutions, which included shelters, drop-in centers, correctional services, and hospitals who were informed about the study and the eligibility criteria for participation. Eligibility criteria for the study were the following: (1) Legal adult status (age 18 or older); (2) housing status of either absolutely homeless (i.e., lacking a regular fixed physical shelter) or precariously housed (living in a rooming house, single-room occupancy unit, or hotel or motel room and having had two episodes or more of homelessness in the past year); (3) the presence of a serious mental disorder (major depressive, manic or hypomanic episode, post-traumatic stress disorder, mood disorder with psychotic features, psychotic disorder) as determined by DSM-IV criteria on the Mini-International Neuropsychiatric Interview (MINI) [27] at the time of study entry; (4) Canadian citizen, landed immigrant, or refugee claimant; and (5) not receiving ACT or ICM at study entry.

Participants were considered to have a high level of need if they were assessed on the MINI as having a diagnosis of a bipolar disorder or psychotic disorder, scored less than 62 on the Multnomah Community Ability Scale (MCAS) [28] and met at least one of the following criteria: (1) Had been hospitalized twice in any one-year period in the past 5 years, (2) had substance abuse or dependence as assessed on the MINI, or (3) had been arrested or incarcerated in the past 6 months. All other participants were considered to have moderate needs.

### Randomization

Randomization was performed by a central data collection system that used an adaptive randomization algorithm to produce equally-sized groups that were balanced on need level. Group assignment was revealed to participants at the end of the first interview. The differences in services received by the two groups precluded blinding, given that data-collection interviews were concerned in part with housing and receipt of services.

### Interventions

#### Housing first

As detailed in the program logic model presented in Table 1, the HF program was based on the Pathways HF approach [6]. Participants received a rent supplement that ensured that they would not contribute more than 30% of their income toward their rent. The housing accessed by HF recipients at the Moncton site was exclusively private market scattered-site rental units. A Housing Coordinator with the program assisted participants in choosing among available units as well as furnishing and moving into them. The Housing Coordinator was also available to mediate if participants encountered difficulties with landlords or neighbors.

In line with the Pathways approach, there were no pre-conditions related to participation in treatment or abstinence from substance use in order to qualify for the HF services. Participants were expected to follow the terms of the lease and make themselves available for one visit per week from program staff. Individualized support was provided to participants by members of a multidisciplinary ACT team. The ACT team operated with a consumer to staff ratio of 10:1, providing services to 100 study participants in the Moncton area. Staff members of the ACT team comprised of a variety of professional disciplines that included a nurse practitioner, psychiatric nurses, an occupational health therapist, a home economist, a social worker, human resource counsellors, a family physician who served as the clinical director, and consulting psychiatrists. The team also added in the later stages of the project two part-time peer support workers who were individuals with lived experience of mental illness and addictions. Additionally, there was an Administrative Manager for the team with training in psychiatric rehabilitation who was available to deliver clinical services to consumers as needed.

An external team made up of Pathways HF staff, the lead researcher of the AHCS trial, and another expert in HF conducted two fidelity assessments, one approximately 10 months after the program began [29] and another when the program was 27 months old [30]. The fidelity assessment entailed visits from three external individuals with expertise in HF who assessed the program on the validated HF with ACT fidelity scale based on observations, interviews with program managers, and focus groups with staff and program participants [29, 30]. Both fidelity assessments showed the program as having good fidelity overall, with 86% of the 37 assessed items rating a score of 3 or more on a 4-point scale in the second fidelity assessment [30].

#### Treatment as usual

Study participants assigned to the TAU group could access all other existing health and social services in the tri-city area. HF services were not available prior to the demonstration project. The region did have some transitional, long-term supportive housing, and non-profit congregate housing as well as social housing units available [24]. A costing study showed that participants in the TAU group consumed on average only a relatively small amount of services from supportive housing programs [23]. Available publicly-funded mental health services were limited to outpatient and day program services in community health centres and hospitals, and psychiatrists in private practice [24]. At the time of the study, there were no ACT or ICM teams delivering services in Moncton.

### Measures

The predicted primary outcomes were housing stability over the study period (i.e., time to first move into stable housing, number of moves, percentage of days housed, perceived housing quality, and being housed consecutively for 6 months or more at the final interview) and community functioning (i.e., level of ability to live independently in the community). The predicted secondary outcomes were self-rated physical and mental health status, substance use, quality of life, community integration, and recovery. The measures for these outcomes were carefully selected based on their history of being used with the population and adequacy of their psychometric properties.

### Primary outcomes

#### Housing stability

The Residential Time-Line Follow-Back Inventory (RTLFB) provides a point-in-time assessment as well as retrospective longitudinal information on housing [31]. Using a calendar, respondents are asked to provide details of their housing for a specific retrospective time period. Specifically, they are asked about the number of moves, the reason for the moves, the type of residence or organization lived in, and the composition of the household. The RTLFB has been shown to have high test-retest reliability and good concurrent validity [31]. In the case of the current study, the RTLFB was administered the first time at the 3-month follow-up asking participants about their housing in the past 6 months in order to determine their housing history just prior to entry into the study. Subsequently, it was administered every 3 months with participants queried about their previous 3 months.

#### Perceived housing quality

The five-item Perceived Housing Quality (PHQL) scale was administered to housed respondents starting at the 6 month follow-up interview [32]. Respondents were asked about the safety, spaciousness, privacy, friendliness, and overall quality of their housing. For example, an item asked “How would you rate your current home for safety?” Response alternatives ranged from 1 (*very bad*) to 5 (*very good*). A summed score on the five items was calculated that can range from 5 to 25. Previous research has shown the measure to have high test-retest reliability [32] and good internal reliability [33]. Cronbach’s alpha for the measure in the current study was 0.88 (6 months), 0.84 (12 months), 0.80 (18 months), and 0.80 (24 months).

#### Community functioning

The 17-item Multnomah Community Ability Scale (MCAS) was used to measure the level of community functioning of participants [28]. The MCAS measures different aspects of community functioning, notably problems in daily functioning as a result of physical and mental and emotional symptoms, ability to cope with mental illness, level of ability to interact with others, and the presence of any behavioural problems that negatively affect living in the community. Based on the information collected in the interview and in response to relevant questions, ratings were completed on each item by interviewers subsequent to each in-person interview. The MCAS has been shown to have good inter-rater reliability, test-retest reliability, and criterion validity (i.e., predictive of hospital admissions for psychiatric problems) [28]. Ratings on the items ranged from *no impairment* (1) to *extreme impairment* (5). The total scale score can range from 17 to 85. Cronbach’s alpha for the MCAS in the current study was 0.77 (baseline), 0.84 (6 months), 0.80 (12 months), 0.82 (18 months), and 0.83 (24 months).

### Secondary outcomes

#### Self-rated health status

Perceived health status was assessed by the EQ-5D, a self-administered 5-item measure that enquires about mobility, self-care, usual activities, pain/discomfort, and anxiety/depression [34]. Each item has three possible responses reflecting no problems, some problems, or extreme problems. A standardized score is calculated across the items that can range from 0 to 2. The EQ-5 has been shown to have good discriminant validity and sensitivity to change in health status [34]. Cronbach’s alpha for the EQ-5D n the current study was 0.70 (baseline), 0.75 (6 months), 0.70 (12 months), 0.72 (18 months), and 0.76 (24 months).

#### Severity of mental health symptoms

The 14-item Colorado Symptom Index (CSI) was used to measure the severity of mental health symptoms [35]. This self-report measure queries the presence and frequency of mental illness symptoms experienced in the past month. Response alternatives can range from 0 (*not at all*) to 4 (*at least every day*) with a possible summed score of 0 to 56. Higher scores reflect a greater severity of symptoms. Research has shown the CSI to have good test-retest reliability, excellent internal consistency, “fair” to “good” discrimination of individuals with psychiatric disabilities, and good convergent and concurrent validity [35]. Cronbach’s alpha for the CSI in the current study was 0.83 (baseline), 0.88 (6 months), 0.87 (12 months), 0.88 (18 months), and 0.88 (24 months).

#### Severity of substance use problems

The 7-item Global Assessment of Individual Need – Substance Problem Scale (GAIN-SPS) was used to measure substance use severity [36]. Participants were queried about when substance-related problems last occurred. The severity of substance use problems was calculated by summing the number of substance-related problems reported in the past month (range 0–5). The GAIN-SPS has been found to have excellent internal consistency, good concurrent validity with objective measures of substance use, and good discriminant validity for detecting the presence of a substance use diagnosis [36]. Cronbach’s alpha for the GAIN-SPS at baseline in the current study was 0.82 (baseline), 0.88 (6 months), 0.87 (12 months), 0.87 (18 months), and 0.88 (24 months).

#### Quality of life

Subjective quality of life was measured using the 20-item Quality of Life Interview (QoLI-20) [37]. Items ask participants about their level of satisfaction with different aspects of their lives. Responses are made on a 7-point Likert scale that ranges from *terrible* (1) to *delighted* (7). The measure includes a total summed score ranging from 20 to 140, and six subscales measuring satisfaction with living situation, finances, leisure, family, social relations, and safety. As well, one item queries overall satisfaction. Higher values correspond to greater quality of life. Internal reliability for the total score and subscales have been shown to be adequate to very good [37]. Cronbach’s alpha for the QoLI-20 in the current study was 0.89 (baseline), 0.91 (6 months), 0.91 (12 months), 0.92 (18 months), and 0.92 (24 months).

#### Physical integration

A 7-item version of a measure of external integration [38] was used to measure level of physical integration. The items asked participants if they had participated in different activities in the community in the past month, such as eating in a restaurant, visiting a library, and participating in a volunteer activity. Possible responses were *yes* (1) or *no* (0). The total summed score on the scale can range from 0 to 7 with higher scores representing relatively higher levels of physical integration. Cronbach’s alpha for the measure in the current study was 0.57 (baseline), 0.57 (6 months), 0.63 (12 months), 0.49 (18 months), and 0.57 (24 months).

#### Psychological integration

A four-item version of a psychological integration measure [39] was used to measure level of community integration. Items asked participants about their sense of belonging associated with where they lived. Response alternatives range from *strongly disagree* (1) to *strongly agree* (5) with possible total summed scores ranging from 4 to 20. A higher summed score corresponded to a higher level of psychological integration. Previous research has found the measure to have acceptable internal reliability [39]. Cronbach’s alpha for the measure in the current study was 0.74 (baseline), 0.73 (6 months), 0.75 (12 months), 0.67 (18 months), and 0.66 (24 months).

#### Recovery

The 22-item Recovery Assessment Scale (RAS) measured the perceived level of recovery from having a severe and persistent mental illness [40]. It was administered at baseline and again at 24 months. Previous research has shown the RAS to be made up of several factors that focus on personal confidence and hope, a willingness to ask for help, goal-setting and future orientation, reliance on others, and no domination by symptoms [40]. Response alternatives vary from *strongly disagree* (1) to *strongly agree* (5). Higher summed scores across the 22 items reflected greater recovery. Possible scores can range from 22 to 110. The RAS has been shown to have excellent internal reliability, high test-retest reliability, and concurrent validity with related measures [40]. Cronbach’s alpha for the RAS in the current study was 0.91 (baseline) and 0.92 (24 months).

### Sample size calculation

The targeted sample size was set at 100 individuals per group. Power analysis determined that a minimum of 63 per group would be sufficient to detect a moderate effect size (ES = 0.50) with α = 0.05 and β = 0.20, allowing for a 35% attrition [15].

### Data collection

In order to maintain contact with participants and collect data on housing history and vocational activities, short interviews were conducted mostly by telephone at 3, 9, 15, and 21 months. Longer interviews were conducted in person and included collecting data on all of the other measures with the exception of the recovery measure at baseline, 6, 12, 18, and 21 or 24 months. In the case of the recovery measure, it was administered at baseline and at the last follow-up interview at 21 or 24 months. The original protocol planned a 24 month follow-up interview. However, the final interviews for a small number of participants (38% of participants who completed the study and 30% of the total sample) were completed at 21 months because of time and resource constraints. At the time of enrolment, participants were invited to provide contact information for themselves and for individuals in their social network who would be likely to know where they are located in the future. As well, participants were asked to give consent for the social services department that administers benefit payments to provide study researchers with their address during the study. These contacts were updated as needed at each follow-up interview.

### Statistical analysis

An intent-to-treat analysis was conducted. Group- and time-specific means for all outcomes were calculated, with 95% confidence intervals produced by bootstrapping with 2000 replications. Linear regression models were used to analyze time to housing and length of stay in housing. For other outcomes, mixed-effects models were used. Linear models were fitted for continuous outcomes, logistic models for binary outcomes, and negative binomial models for counts of events. In all cases sex, age, and level of need (i.e., moderate or high) were entered as covariates.

Mixed effects models were random-intercept models. Time was treated as a categorical variable by dummy-coding events and interacting each time point with group membership, producing an expected mean and an expected group difference at each time point. Two measures of group differences were produced: differences between groups at the final time point, and the average difference over all the follow-up events (representing differences over the study period as a whole).

Secondary analyses examined group*need level interactions. Due to the large number of interaction terms that would otherwise be necessary, we fit simplified mixed effects models in which outcomes were regressed on their baseline level, group, need, group*need, and covariates. In these models, coefficients for group*need interactions represent overall intervention effect differences over the follow-up period as a whole.

In order to examine for any difficulties associated with missing data, a sensitivity analysis was conducted with a set of 40 imputed data sets for community functioning (i.e., MCAS) developed with sequential regression multivariate imputation. This analysis produced an effect very similar to that of the original model, and no meaningful differences in covariates (e.g., in terms of significance or non-significance). For housing, which was the other primary outcome, the large effects and the relatively small amount of missing data essentially rule out any effects of missing data on inferences.

## Results

### Study participants

**Fig. 1:**
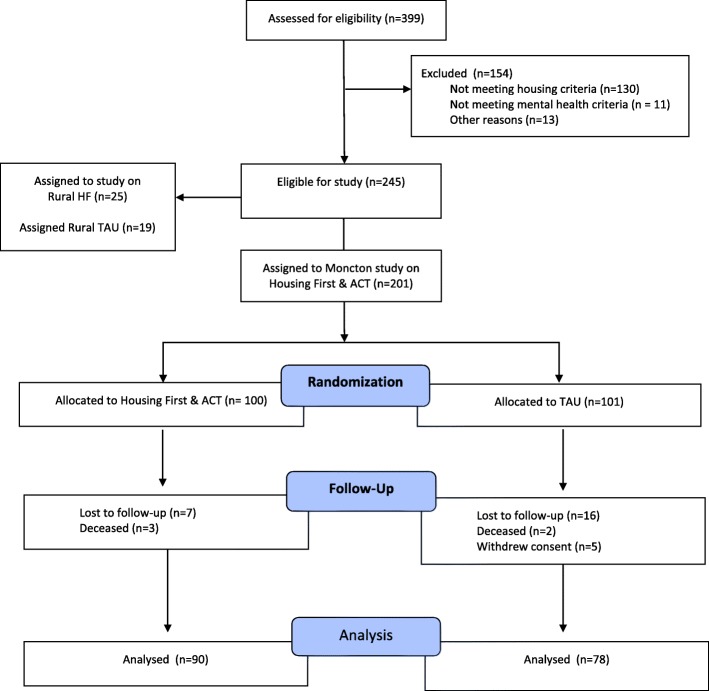
Screening, Randomization, and Follow-up of Participants

A CONSORT diagram of the screening, randomization, and follow-up of participants is presented in Fig. 1. Baseline interviews began in October 2009 and ended in April 2011, with the last follow-up interview conducted in February 2013. A total of 168 (84%) individuals completed the last follow-up interview at 21 or 24 months, comprising of 90 of 101 (89%) HF participants and 75 of 100 (75%) TAU participants. Although all of the sites showed greater attrition in the TAU group, a detailed analysis of participants in the AHCS project lost at the last follow-up found no differences between the groups on their demographic or clinical characteristics [41]. Moreover, there were some variables that predicted attrition (date of enrolment, substance use disorder, and psychotic disorder, as well as site) but the effects were small and they did not differ significantly between HF and TAU. Table 2 presents the characteristics of the participants at baseline.

**Table 2:** Characteristics of Study Participants^a^

	Housing First		Treatment As Usual	
	*n*	%	*n*	%
Gender				
Male	67	66	63	63
Female	33	33	37	37
Trans	1	1	0	0
Age				
18–30	29	29	22	22
31–40	21	21	27	27
41–50	31	31	37	37
51+	20	20	14	14
Language				
English	75	74	66	66
French	24	24	31	31
Other	2	2	3	3
Ethnocultural identity				
Indigenous	6	6	3	3
White	80	79	82	82
Other	15	15	15	15
Education				
Grade 8 or less	15	15	21	21
Some high school	42	42	38	38
Completed high school	21	21	24	24
Postsecondary	22	22	17	17
Marital status				
Single	67	66	66	66
Sep., div., widow	31	31	28	28
Married or common law	3	3	6	6
Housing status				
Absolutely homeless	54	54	59	59
Precariously housed	47	47	41	41
Lifetime homelessness (months)				
0–12	37	37	33	34
13–24	14	14	15	16
24–36	9	9	15	16
37–48	8	8	13	13
48+	31	31	21	22
Need level				
High need	35	35	38	38
Moderate need	66	65	62	62
Mental health				
Major depressive episode	66	65	76	76
Manic or hypomanic episode	18	18	16	16
PTSD	44	44	47	47
Panic disorder	27	27	29	29
Mood disorder (psych. feats.)	12	12	14	14
Psychotic disorder	22	22	25	25
Addictions				
Alcohol dependence	31	31	32	32
Substance dependence	56	55	51	51
Chronic health conditions				
0–1	12	12	9	9
2–4	31	31	28	28
5–8	37	37	43	43
8+	20	20	20	20

^a^There were no significant differences between the baseline characteristics of the groups

### Primary outcomes

Table 3 presents results for the primary outcomes. On average, among participants who were absolutely homeless at study entry and eventually became housed, HF participants moved into housing much more rapidly than TAU participants (23.30 versus 88.25 days, *d* = 1.02, 95% CI 0.50–1.53], *p* = 0.001). For both groups, the proportion meeting our definition of ‘stably housed’ rose within the first 6 months of follow-up. For the intervention group, this proportion increased rapidly from 25 to 90% after only 3 months before plateauing at just below 90%. In the control group, it rose from 17% at baseline to 27% after 3 months and 41% after 6 months, and then stabilized at 42–47% for the rest of the follow-up period. On average, controlling for differences at baseline, the intervention group was about three times as likely as the control group to be stably housed (*Z* = 5.30, *p* < 0.001, *OR* = 3.12, 95% CI [1.96–4.27]).

**Table 3:** Means and Standard Deviations of Primary Outcomes

	Housing First		Treatment as Usual	
	(*N* = 72–101)		(*N* = 51–100)	
	M	SD	M	SD
Days to becoming housed (RTFLB)	23.30	17.91	88.25	88.55
% time housed (RTFLB)				
Baseline	25.71	39.51	16.13	36.44
3 months	84.39	26.12	25.98	40.04
6 months	90.64	24.74	39.90	44.22
9 months	89.84	27.24	42.37	46.59
12 months	87.58	29.06	43.57	46.75
15 months	90.47	26.12	43.41	46.44
18 months	87.04	30.43	46.96	47.14
21 months	86.79	29.58	44.08	45.62
Final follow-up	87.73	29.66	47.84	47.38
# of moves (RTFLB)	2.87	2.33	5.47	5.10
Housed 6 mos. + (RTFLB)	79.57%		55.84%	
Housing qual. (PHQL)^a^				
6 months	20.73	3.66	16.79	6.17
12 months	20.75	3.67	17.47	5.13
18 months	19.88	4.02	19.85	4.44
21/24 months	20.13	3.84	18.56	3.75
Comm. funct. (MCAS)^b^				
Baseline	59.45	8.44	58.26	7.99
6 months	63.51	9.15	62.69	8.57
12 months	65.45	8.10	63.62	6.77
18 months	65.66	7.70	65.04	9.01
21/24 months	64.75	8.25	64.27	8.85

^a^PHQL scores can range from 5 to 25, with higher scores reflecting a higher level of the perceived quality of housing

^b^MCAS scores can range from 17 to 85, with higher scores reflecting a higher level of community functioning

In line with demonstrating greater housing stability, HF participants experienced fewer moves than TAU participants over the course of the study (2.87 versus 5.47, *d* = 0.67, 95% CI [0.36–0.97], *p* < 0.001). HF participants were also more likely than TAU participants to be housed *all of the time* in the final 6 months of the study (79.6% vs. 55.5%; χ^2^ (2, *n* = 170) = 11.46, *p* = 0.003, Cramer’s V = 0.26, 95% CI [0.14–0.42]). Perceived housing quality (PHQL) for housed participants remained fairly stable from six to 24 months in the HF group (there was no baseline measure for this variable, as participants at baseline were either relatively or absolutely homeless). In TAU, PHQL scores increased over the course of the study but never exceeded those in the intervention group. The average difference during follow-up between the two groups was statistically significant, with HF participants assessing the quality of their housing as better than TAU participants (*Z* = 4.59, *p* < 0.001, *d* = 0.43, 95% CI [0.25–0.62]).

Community functioning, as measured by the MCAS, increased significantly in both groups over the course of the study (*Z* = 10.41*, p* < 0.001, pooled ASMD = 0.65, 95% CI [0.53–0.77]). However, no significant differences between groups were observed.

### Secondary outcomes

Table 4 presents results for the secondary outcomes. Both groups showed similarly significant improvements in self-reported health status (*Z* = 5.03*, p* < 0.001, pooled ASMD = 0.33, 95% CI [0.20–0.46]), severity of mental health symptoms (*Z* = 11.06*, p* < 0.001, pooled ASMD = 0.76, 95% CI [0.63–0.90]), and substance use problems (*Z* = 4.90*, p* < 0.001, pooled ASMD = 0.38, 95% CI [0.23–0.54]). Although both groups reported significant changes in quality of life over the course of the study (*Z* = 15.79*, p* < 0.001 pooled ASMD = 1.04, 95% CI [0.91–1.17]), HF participants demonstrated greater improvements than TAU participants (*Z* = 3.83, *p* < 0.001, ASMD = 0.50, 95% CI [0.24–0.75]).

**Table 4:** Means and Standard Deviations of Secondary Outcomes

	Housing First		Treatment as usual	
	(*N* = 63–101)		(*N* = 53–100)	
	M	SD	M	SD
Quality of life (QoLI-20)^a^				
Baseline	63.79	17.69	64.76	20.55
21/24 months	88.92	19.27	81.70	24.38
Physical Integration^b^				
Baseline	1.76	1.39	1.87	1.73
21/24 months	2.09	1.40	1.95	1.87
Psychological Integration^c^				
Baseline	8.99	3.56	9.30	4.05
21/24 months	12.91	2.94	11.90	3.75
Physical health (EQ. 5D)^d^				
Baseline	0.45	0.22	0.45	0.22
21/24 months	0.37	0.24	0.37	0.25
Psych. symptoms (CSI)^e^				
Baseline	41.74	9.35	43.89	9.22
21/24 months	32.92	12.03	34.78	11.68
Subst. use probl. (GAIN)^f^				
Baseline	1.84	1.70	1.90	1.79
21/24 months	1.24	1.69	1.23	1.74
Recovery (RAS)^g^				
Baseline	76.00	12.67	74.27	15.22
21/24 months	89.11	10.74	84.21	11.14

^a^QOL-20 scores can range from 20 to 140 with higher scores reflecting a higher quality of life

^b^Scored can range from 0 to 7 with higher scores reflecting a higher level of physical integration

^c^Scores can range from 4 to 20, with higher scores reflecting a higher level of psychological integration

^d^EQ 5D scores can range from 0 to 1, with higher scores reflecting better perceived health status

^e^CSI scores can range from 0 to 56, with higher scores reflecting more severe mental illness symptoms

^f^GAIN scores can range from 0 to 5, with higher scores reflecting more symptoms of substance misuse in past month

^g^RAS scores can range from 22 to 110, with higher scores reflecting a higher level of perceived recovery

An examination of results on the quality of life measure subscales showed HF participants reported greater improvements in the areas of living situation (*Z* = 5.69, *p* < 0.001, ASMD = 1.04, 95% CI [0.68–1.40]), safety (*Z* = 3.54, *p* < 0.001, ASMD = 0.48, 95% CI [0.22–0.75]), finances (*Z* = 2.90, *p* = 0.004, ASMD = 0.46, 95% CI [0.15–0.77]), and leisure (*Z* = 2.57, *p* = 0.01, ASMD = 0.34, 95% CI [0.08–0.60]). Both groups experienced similarly significant changes in the quality of life areas of family (*Z* = 7.17, *p* < 0.001, pooled ASMD = 0.46, 95% CI = [0.33–0.59]) and social relations (*Z* = 6.83, *p* < 0.001, pooled ASMD = 0.45, 95% CI = [0.32–0.58]).

Both groups showed no change in physical integration and significant improvements in psychological integration (*Z* = 12.89, *p* < 0.001, pooled ASMD = 0.91, 95% CI [0.77–1.05]). However, HF participants reported greater improvements in psychological integration than TAU participants (*Z* = 2.35, *p* = 0.02, ASMD = 0.33, 95% CI [0.05–0.61]). Both groups reported improvements in their level of recovery (*Z* = 6.55, *p* < 0.001, pooled ASMD = 0.81, 95% CI [0.56–1.06]), although HF participants showed greater improvement than TAU participants (*Z* = 2.26, *p* = 0.03, ASMD = 0.39, 95% CI [0.05–0.74]). Analyses of subscales of the RAS found differences in level of improvement in the area of reliance on others, with HF participants experiencing greater improvement in this area than TAU participants (*Z* = 2.27, *p* = 0.03, ASMD = 0.33, 95% CI [0.04–0.61]).

The examination of the interaction of group and level of need found no significant interaction for the primary outcomes and for any secondary outcomes with the exception of quality of life. HF was associated with smaller improvements for high-need participants compared to moderate-need participants in overall quality of life (*p* = 0.01, ASMD = 0.61, 95% CI [0.14–1.08]), quality of life associated with social relations (*p* = 0.03, ASMD = 0.35, 95% CI [0.04–0.66]), and quality of life related to family relations (*p* = 0.03, ASMD = 0.61, 95% CI [0.07–1.15]).

## Discussion

Our study demonstrates that the delivery of HF with ACT in a small city produces similarly superior housing outcomes to standard care as previously found in large American and Canadian cities [5, 10–13, 20]. HF participants exited homelessness more rapidly, spent a greater proportion of time in stable housing, experienced fewer moves, and were much more likely to be housed consecutively for 6 months or more at the end of the study in comparison to TAU participants. The effect size on all the tested housing outcomes was very large in the direction favoring the HF group, ranging from *d* = 1.02 for being housed more rapidly to *OR* = 3.12 of being more likely to be stably housed.

In addition, HF participants rated their housing in terms of safety, spaciousness, privacy, friendliness, and overall quality as being better than TAU participants. It is likely that the rent supplement provided to HF participants enabled them to access better housing. As well, it is possible that the choice provided to HF participants in selecting housing also contributed to them perceiving their housing as being of better quality. Previous research in which rent supplements were provided to individuals and combined with ICM also yielded superior housing quality, more positive housing characteristics, and fewer negative characteristics for individuals receiving HF compared to standard care [42, 43].

Both groups showed similar levels of improvements in community functioning over the course of the study. Although most participants in the control group were also receiving non-study services, some improvement is also likely to be due to regression to the mean. Some people may have been recruited to the study when in crisis, and may then have experienced some degree of natural improvement; this view is consistent with the waxing and waning of symptom severity associated with chronic mental illnesses [44]. It is also conceivable that the study included a few people whose real level of functioning was somewhat higher than study criteria specified, either through ordinary measurement error or because of participants’ desire to ensure they met those criteria, and that follow-ups simply captured their true level of functioning.

Although community functioning improved substantially in both groups, we did not observe an intervention effect. The full multi-site study, with a much larger sample size, did observe a small benefit for the intervention in this area [19]. In Moncton, however, our results imply an effect produced by HF close to zero, and exclude any large benefit (ASMD = − 0.02, 95% CI = − 0.26 to 0.23 for the average of post-baseline follow-ups, and ASMD = − 0.09, 95% CI = − 0.40 to 0.22 for the final time-point). This suggests that the provision of community support in the context of housing stability does not achieve, at least in a two-year period, different outcomes in this area when compared to the range of services accessed by TAU participants. Previous research on HF using the Pathways approach has not examined community functioning as an outcome.

In line with these latter findings, both groups also reported similar levels of improvement in terms of self-reported health status, severity of mental health symptoms, and number of problems related to substance use. These improvements ranged from demonstrating small effects (self-reported health status and number of problems related to substance use) to large effects (severity of mental health symptoms). Again, improvements may be the result of regression to the mean, with participants entering the study at a particularly difficult point, and returning, on average, to a higher level of functioning over time. As well, improvements for both groups on health-related outcomes are likely to be at least in part a response to the health and social services that they received over the course of the study. Curiously, although HF recipients as a group were found to have more days of hospitalizations for psychiatric difficulties [23], this did not appear to produce better health outcomes than TAU. As previously mentioned, these findings were explained as being the result of support provided by the ACT team who were able to help participants access psychiatric hospital treatment when it was needed [22].

Although both groups reported improvements in overall quality of life, HF participants experienced significantly greater outcomes in this area compared to TAU participants. An examination of specific areas of quality of life found HF participants reporting greater improvements in relation to their living situation, safety, finances, and leisure. It is a plausible interpretation that these results are associated with a high proportion of HF participants achieving housing stability over the course of the study. As well, the rent subsidy provided to HF participants can be expected to enable individuals to afford a better and safer living situation as well as some additional financial resources for engaging in leisure activities.

Previous studies have shown HF with ACT to yield better quality of life outcomes compared to standard care globally and in the areas of living situation and safety [20] as well as other areas including leisure, health, daily activities, social relationships, and family relationships [13]. The multi-city trial in which the current study was conducted also found HF with ACT, when compared to TAU for individuals with high level of needs, had better overall quality of life outcomes and in the same specific areas (i.e., living situation, personal safety, leisure activities) after 12 months [18] but not at 24 months [19].

On the other hand, in the same trial, HF with ICM, when compared to TAU for individuals with moderate level of needs, produced greater improvements in overall quality of life and related to living situation, safety, and leisure [45]. In our study, HF participants with a mix of moderate and high level of needs and receiving ACT reported greater improvements of quality of life than TAU over the full 24-month study. This finding is an important contribution that goes above and beyond the multi-site study findings.

At the same time, individuals with moderate level of needs in our study experienced greater improvements in overall quality of life and in relation to family relations and social relations than individuals with high level of needs. These findings combined with those from the multi-site study suggest that individuals with less severe difficulties and lower level of needs receiving HF are more prone to experiencing enhanced quality of life in the context of, and possibly as a result of, having achieved housing stability.

It is important to note that the differentiation of individuals with moderate level of needs from those with high level of needs included them being diagnosed with a serious mental disorder other than psychotic disorder or bipolar disorder (i.e., major depression, post-traumatic stress disorder). It is possible that these diagnostic differences are contributing to these greater improvements in subjective quality of life for individuals with moderate needs.

Both groups also demonstrated improvements in physical integration and psychological integration over the course of the study. In addition, HF participants reported greater improvements in psychological integration than TAU participants. A fundamental standard of the Pathways HF approach entails giving individual’s choice over their housing including its location [6]. This choice may contribute to HF participants living in housing and neighborhoods in which they feel at home and develop a sense of belonging. In contrast with our study, the multi-site study did not show HF participants experiencing greater improvements in psychological integration than TAU for either individuals with high needs [19] or moderate needs [45]. Previous research has shown residents living in small cities to report higher levels of a sense of belonging than residents living in larger cities [46]. In this context, it is possible that being stably housed in smaller cities like Moncton provides the conditions for recipients of HF to experience greater improvements in psychological integration than those accessing TAU.

Another unique finding emerging from our study concerned perceived level of recovery from having a serious mental illness. Similar to many of the other non-housing outcomes in our study, both groups reported perceiving improvements in overall recovery and in terms of the specific areas making up overall recovery, namely experiencing enhanced self-confidence and hope, an increased willingness to ask for help, improved goal-setting abilities, increased comfort with relying on others for assistance, and diminished sense of being dominated by symptoms.

At the same time, HF participants reported greater improvement in their overall perceived level of recovery and in their reliance on others compared to TAU participants. These findings represent the first time that HF has been shown to produce superior outcomes in this area. They suggest that the housing stability and support provided through HF assisted individuals to become more future- and goal-oriented as well as increased their willingness to ask for assistance and rely on others.

Strengths of our study include the low attrition rate, the results of two program assessments showing the achievement of high fidelity to the approach, and the examination of a wide range of housing and non-housing outcomes. Limitations of the study include the non-blinding of the study, the greater attrition of TAU participants, the relatively short length of follow-up, the heavy reliance on self-report measures of outcomes such as subjective quality of life that may be influenced by the clinical state of participants, and the low internal reliability of the physical integration measure. The reliance on the MINI to identify the presence of specific diagnoses for determining eligibility in the study and for differentiating individuals with high needs versus moderate needs is also a noteworthy limitation.

## Conclusions

Our study extends the research findings on HF by evaluating its effectiveness in the context of a small city that offers traditional hospital-based inpatient and outpatient psychiatric services as well as limited and largely office-based community based mental health services [24]. In this context, HF was shown to be effective in producing superior housing outcomes as well as better non-housing outcomes, notably in relation to quality of life, psychological integration, and perceived recovery. Future research should involve investigating HF in other smaller cities to determine if the additional positive psychosocial benefits are replicated. As well, it would be worthwhile to follow HF recipients for a longer period of time to determine if these benefits are sustained and if they experience other areas of improvement related to being stably housed.

## Data Availability

The datasets used and/or analysed during the current study and the interview protocols are available from the corresponding author upon reasonable request.

## Abbreviations

ACT: Assertive community treatment
AHCS: At Home / Chez Soi
HF: Housing First
MCAS: Multnomah Community Ability Scale
MINI: Mini international neuropsychiatric interview
TAU: Treatment as usual

## References

[1] Gaetz S, Dej E, Richter T, Redman M (2016). The State of Homelessness in Canada 2016.

[2] Aubry T, Klodawsky F, Coulombe D (2012). Comparing the housing trajectories of different classes within a diverse homeless population. Am J Community Psychol.

[3] Fazel S, Khosla V, Doll H, Geddes J (2008). The prevalence of mental disorders among the homeless in western countries: systematic review and meta-regression analysis. PLoS Med.

[4] Kuhn R, Culhane D (1998). Applying cluster analysis to test a typology of homelessness by pattern of shelter utilization: results from the analysis of administrative data. Am J Community Psychol.

[5] Tsemberis S (1999). From streets to homes: an innovative approach to supported housing for homeless adults with psychiatric disabilities. J Community Psychol.

[6] Tsemberis S (2010). Housing First: the pathways model to end homelessness for people with mental illness and addiction.

[7] Aubry T, Nelson G, Tsemberis S (2015). Housing First for people with severe mental illness who are homeless: a review of the research and findings from the At Home-Chez soi demonstration project. Can J Psychiatr.

[8] Rog DJ, Marshall T, Dougherty R, George P, Daniels AS, Ghose SS, Delphin-Rittmon ME (2014). Permanent supportive housing: assessing the evidence. Psychiatr Serv.

[9] Gulcur L, Tsemberis S, Stefancic A, Greenwood RM (2007). Community integration of adults with psychiatric disabilities and histories of homelessness. Community Ment Health J.

[10] Stefancic A, Tsemberis S (2007). Housing First for long-term shelter dwellers with psychiatric disabilities in a suburban county: a four-year study of housing access and retention. J Prim Prev.

[11] Tsemberis S, Eisenberg RF (2000). Pathways to housing: supported housing for street-dwelling homeless individuals. Psychiatr Serv.

[12] Appel PW, Tsemberis S, Joseph H, Stefancic A, Lambert-Wacey D (2012). Housing First for severely mentally ill homeless methadone patients. J Addict Dis.

[13] Gilmer TP, Stefancic A, Ettner SL, Manning WG, Tsemberis S (2010). Effect of full-service partnerships on homelessness, use and costs of mental health services, and quality of life among adults with serious mental illness. Arch Gen Psychiatry.

[14] Padgett DK, Stanhope V, Henwood BF, Stefancic A (2011). Substance use outcomes among homeless clients with serious mental illness: comparing Housing First with treatment first programs. Community Ment Health J.

[15] Goering PN, Streiner DL, Adair C, Aubry T, Barker J, Distasio J (2011). The At Home/Chez Soi trial protocol: a pragmatic, multi-site, randomized controlled trial of a Housing First interventions for homeless individuals with mental illness in five Canadian cities. Brit Med J Open.

[16] Nelson G, Stefancic A, Rae J, Townley G, Tsemberis S, Mcnaughton E (2014). Early implementation evaluation of a multi-site Housing First intervention for homeless people with mental illness: a mixed methods approach. Eval Program Plann.

[17] Mcnaughton E, Stefanic A, Nelson G, Caplan R, Townley G, Aubry T (2015). Implementing Housing First across sites and over time: later Fidelity and implementation evaluation of a pan-Canadian multi-site Housing First program for homeless people with mental illness. Am J Community Psychol.

[18] Aubry T, Tsemberis S, Adair CE, Veldhuizen S, Streiner D, Latimer E (2015). One-year outcomes of a randomized controlled trial of Housing First with ACT in five Canadian cities. Psychiatr Serv.

[19] Aubry T, Goering P, Veldhuizen S, Adair CE, Bourque J, Disatsio J (2016). A multiple city RCT of Housing First with assertive community treatment for homeless Canadians with serious mental illness. Psychiatr Serv.

[20] Patterson M, Moniruzzaman A, Palepu A, Zabkiewicz D, Frankish CJ, Krausz M (2013). Housing First improves subjective quality of life among homeless adults with mental illness: 12-month findings from a randomized controlled trial in Vancouver. British Columbia Soc Psychiat Psychiatr Epidemiol.

[21] Somers JM, Rezansoff SN, Moniruzzaman A, Palepu A, Patterson M (2013). Housing First reduces re-offending among formerly homeless adults with mental disorders: Results of a randomized controlled trial. PLoS One.

[22] Pakzad S, Bourque P-E, Bourque J, Aubry T, Gallant L, LeBlanc SR (2017). A comparison of the use of physical and mental health services by homeless people with severe mental health problems in the Moncton area through the At Home/Chez Soi program. Can J Commun Ment Health.

[23] Latimer Eric A., Rabouin Daniel, Cao Zhirong, Ly Angela, Powell Guido, Aubry Tim, Distasio Jino, Hwang Stephen W., Somers Julian M., Stergiopoulos Vicky, Veldhuizen Scott, Moodie Erica E.M., Lesage Alain, Goering Paula N. (2017). Costs of services for homeless people with mental illness in 5 Canadian cities: a large prospective follow-up study. CMAJ Open.

[24] Health Systems Research and Consulting Unit (2009). A Review of the Structure and Delivery of Community Mental Health Services in New Brunswick: Final Report.

[25] Statistics Canada; Census of Canada 2016. Information on Moncton. https://bit.ly/2N8d30p . Accessed 15 Feb 2019.

[26] Greater Moncton Homelessness Steering Committee (2010). Experiencing Homelessness: The third report card on homelessness in Greater Moncton.

[27] Sheehan DV, Lecrubier Y, Harnett-Sheehan K, Amorim P, Janavs J, Weiller E (1998). The Mini International Neuropsychiatric Interview (M.I.N.I.): The development and validation of a structured diagnostic psychiatric interview. J Clin Psychiat.

[28] Barker S, Barron N, McFarland BH, Bigelow DA (1994). A community ability scale for chronically mentally ill consumers. Community Ment Health J.

[29] Ecker J, Aubry T, Cherner R, Jetté J (2014). Implementation evaluation of a Housing First program in a small Canadian city. Can J Comm Ment Health.

[30] Aubry T, Ecker J, Yamin S, Jette J, Sylvestre J (2015). Findings from a fidelity assessment of a Housing First programme in a small Canadian city. Eur J Homelessn.

[31] Tsemberis S, McHugo G, Williams V, Hanrahan P, Stefancic A (2007). Measuring homelessness and residential stability: the residential time-line follow-back inventory. J Community Psychol.

[32] Toro PA, Passero Rabideau JM, Bellavia CW, Daeschler CV, Wall DD, Thomas DM (1997). Evaluating an intervention for homeless person: results of a field experiment. J Consult Clin Psychol.

[33] Aubry T, Duhoux A, Klodawsky F, Ecker J, Hay E (2016). A longitudinal study of predictors of housing stability, housing quality, and mental health functioning among single homeless individuals staying in emergency shelters. Am J Community Psychol.

[34] Lamers LM, Bouwmans CAM, van Straten A, Donker CH, Hakkaart L (2006). Comparison of EQ-5D and SF-6D utilities in mental health patients. Health Econ.

[35] Boothroyd RA, Chen HJ (2008). The psychometric properties of the Colorado symptom index. Adm Policy Ment Hlth.

[36] Dennis ML, Chan YF, Funk RR (2006). Development and validation of the GAIN short screener for internalizing, externalizing and substance use disorders and crime violence problems among adolescents and adults. Am J Addict.

[37] Lehman AF (1996). Measures of quality of life among persons with severe and persistent mental disorders. Soc Psychiatry Psychiatr Epidemiol.

[38] Segal SP (1978). Aviram U the mentally ill in community based sheltered care: a study of community care and social integration.

[39] Aubry T, Myner J (1996). Community integration and quality of life: a comparison of persons with psychiatric disabilities in housing programs and community residents who are neighbors. Can J Commun Ment Health.

[40] Corrigan PW, Salzer M, Ralph RO, Sangster Y, Keck L (2004). Examining the factor structure of the recovery assessment scale. Schizophr Bull.

[41] Veldhuizen S, Adair CE, Methot C, Kopp BC, O’Campo P, Bourque J (2014). Patterns and predictors of attrition in a trial of a housing intervention for homeless people with mental illness. Soc Psychiatry Psychiatr Epidemiol.

[42] Cheng A, Lin H, Kasprow W, Rosenheck RA (2007). Impact of supported housing on clinical outcomes: analysis of a randomized trial using multiple imputation technique. J Nerv Ment Dis.

[43] Rosenheck R, Kasprow W, Frisman L, Liu-Mares W (2003). Cost-effectiveness of supported housing for homeless persons with mental illness. Arch Gen Psychiatry.

[44] Finney JW (2008). Regression to the mean in substance abuse treatment research. Addiction..

[45] Stergiopoulos V, Gozdzik A, Misir V, Skosireva A, Sarang A, Connelly J (2016). The effectiveness of a Housing First adaptation for ethnic minority groups: findings of a pragmatic randomized controlled trial. BMC Public Health.

[46] Kitchen P, Williams AM, Gallina M (2015). Sense of belonging to local community in small-to-medium sized Canadian urban areas: a comparison of immigrant and Canadian-born residents. BMC Psychol.

